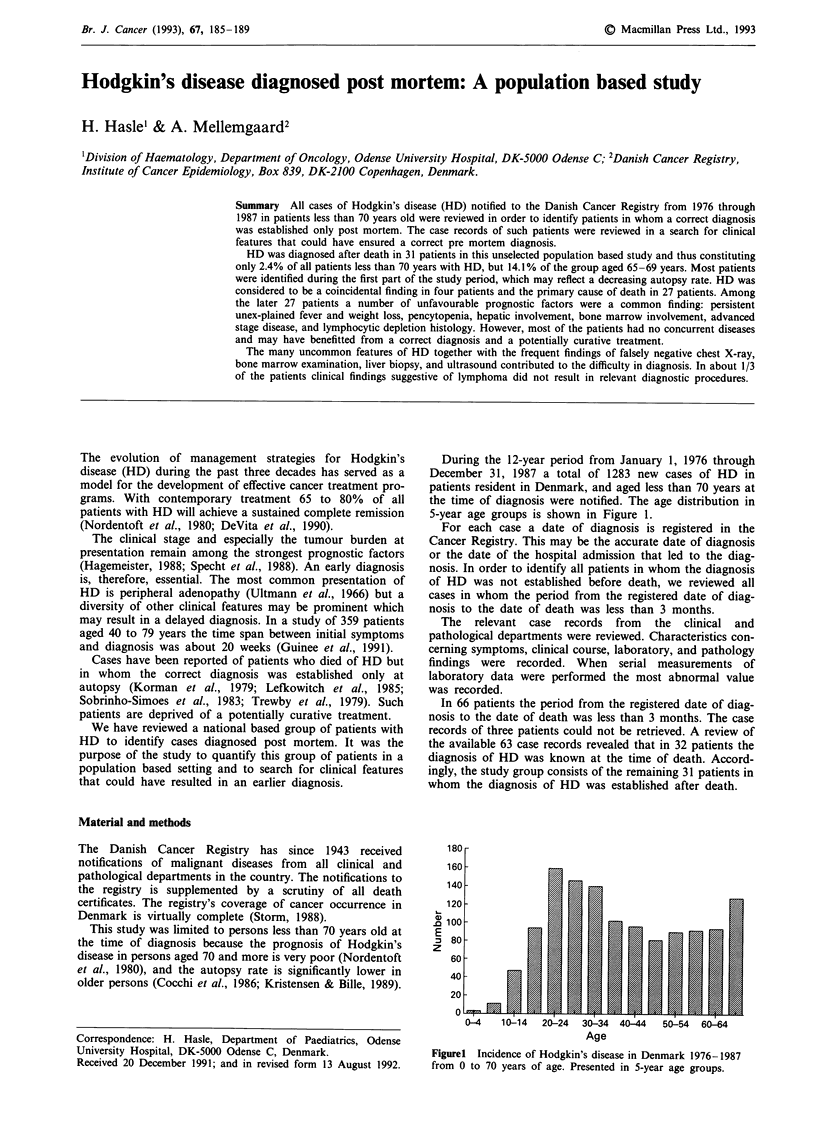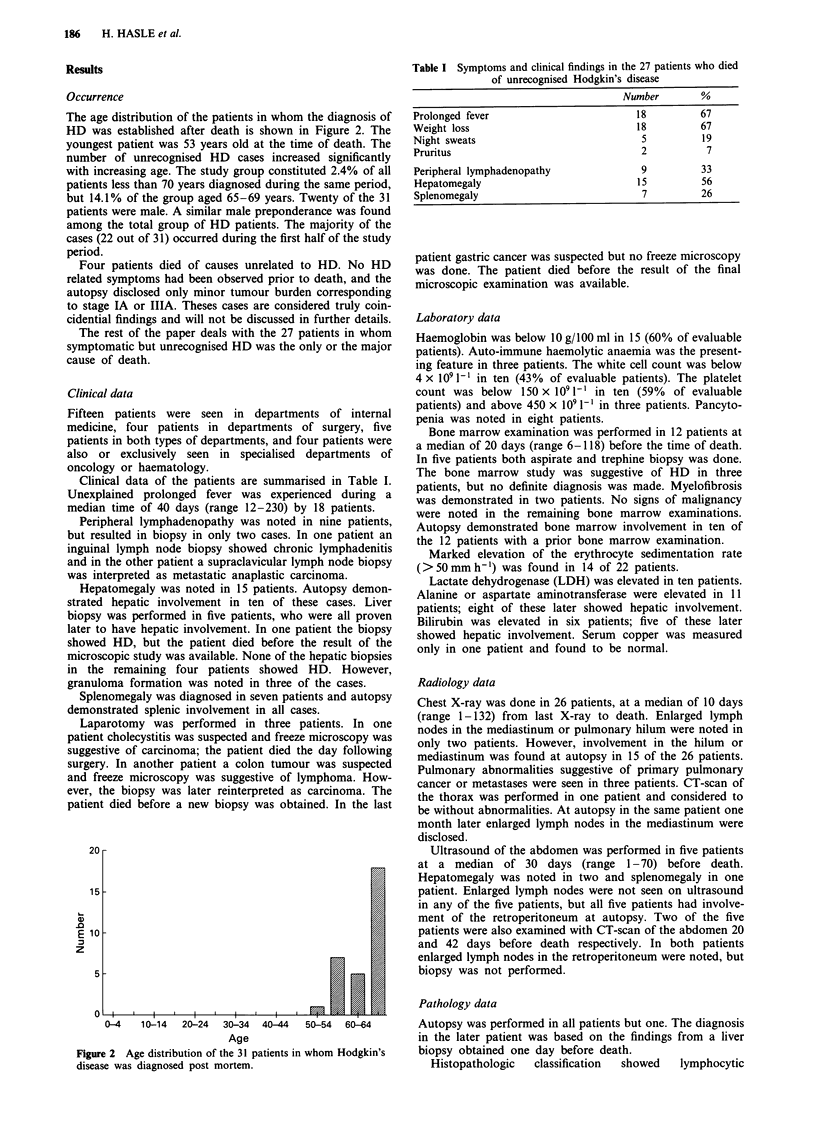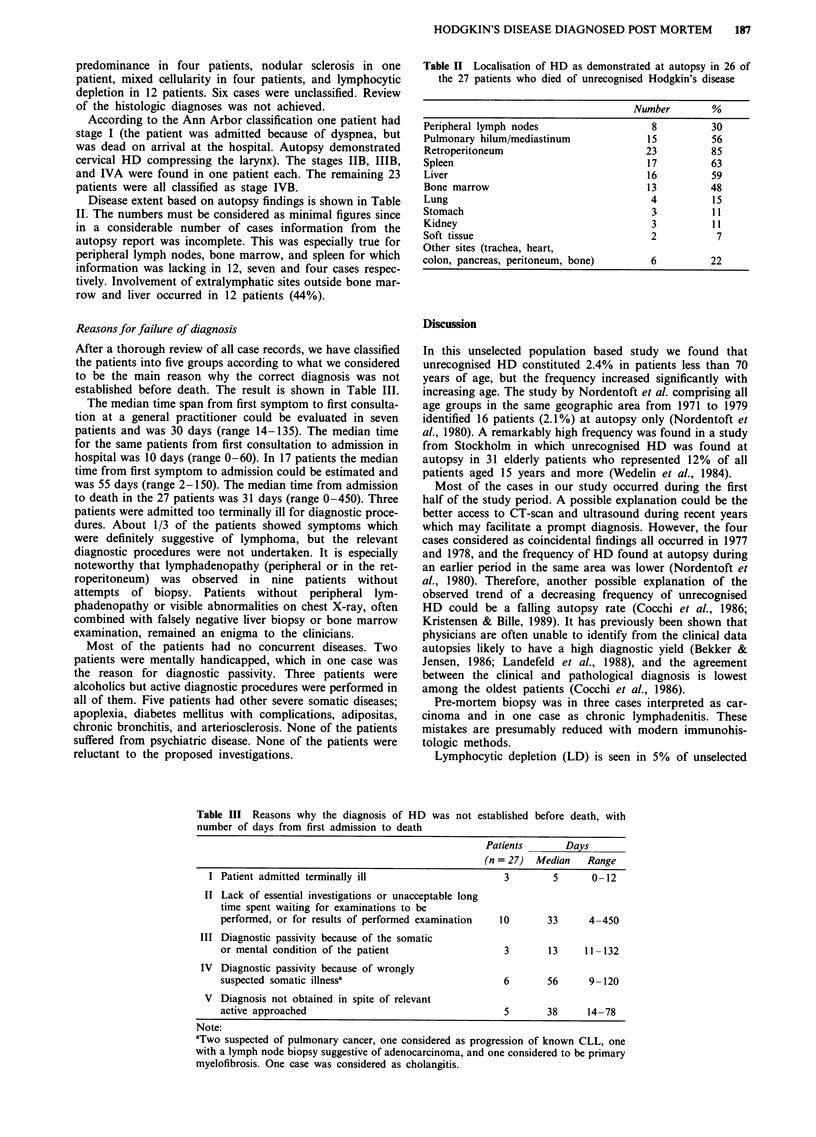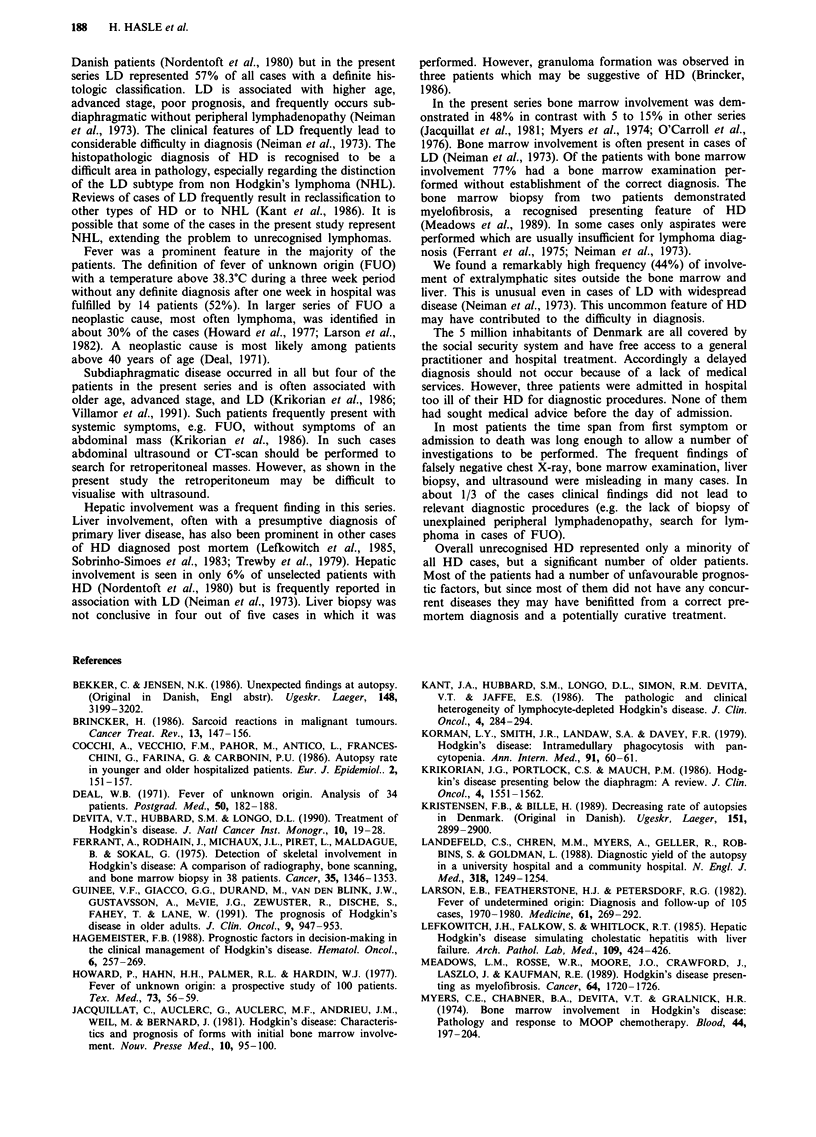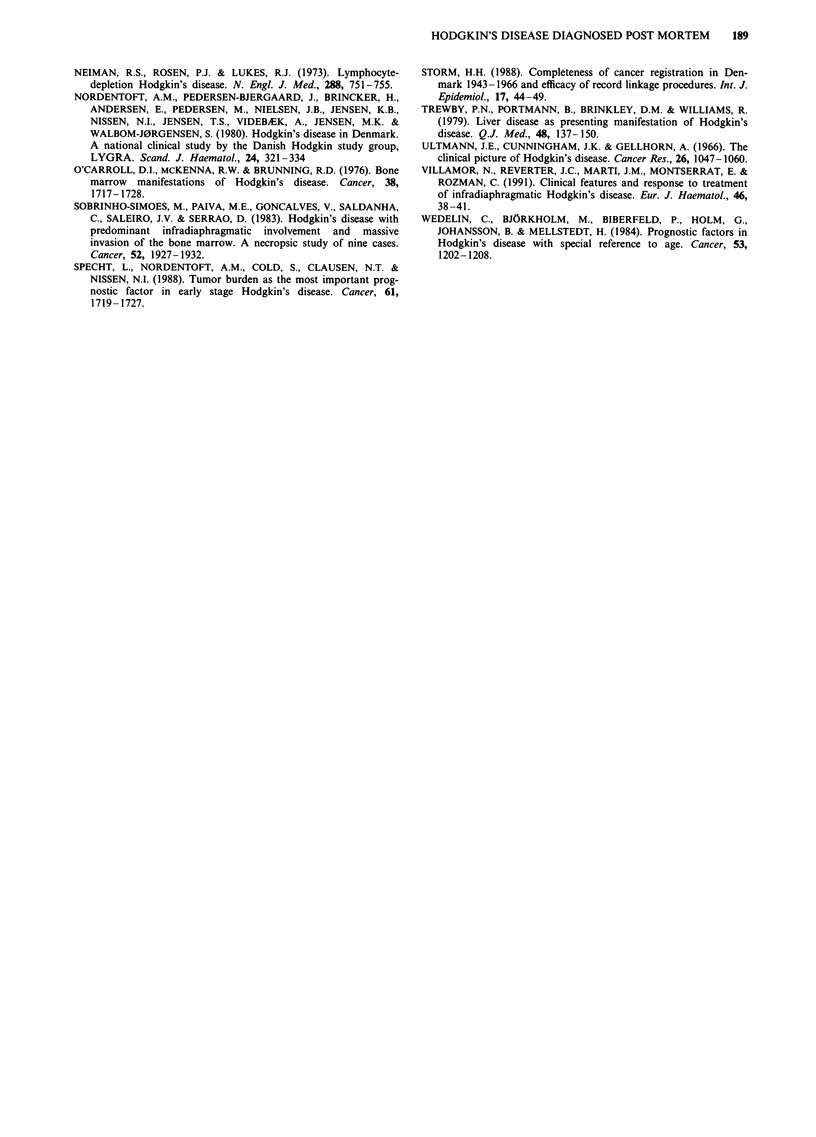# Hodgkin's disease diagnosed post mortem: a population based study.

**DOI:** 10.1038/bjc.1993.32

**Published:** 1993-01

**Authors:** H. Hasle, A. Mellemgaard

**Affiliations:** Department of Oncology, Odense University Hospital, Denmark.

## Abstract

All cases of Hodgkin's disease (HD) notified to the Danish Cancer Registry from 1976 through 1987 in patients less than 70 years old were reviewed in order to identify patients in whom a correct diagnosis was established only post mortem. The case records of such patients were reviewed in a search for clinical features that could have ensured a correct pre mortem diagnosis. HD was diagnosed after death in 31 patients in this unselected population based study and thus constituting only 2.4% of all patients less than 70 years with HD, but 14.1% of the group aged 65-69 years. Most patients were identified during the first part of the study period, which may reflect a decreasing autopsy rate. HD was considered to be a coincidental finding in four patients and the primary cause of death in 27 patients. Among the later 27 patients a number of unfavourable prognostic factors were a common finding: persistent unexplained fever and weight loss, pancytopenia, hepatic involvement, bone marrow involvement, advanced stage disease, and lymphocytic depletion histology. However, most of the patients had no concurrent diseases and may have benefitted from a correct diagnosis and a potentially curative treatment. The many uncommon features of HD together with the frequent findings of falsely negative chest X-ray, bone marrow examination, liver biopsy, and ultrasound contributed to the difficulty in diagnosis. In about 1/3 of the patients clinical findings suggestive of lymphoma did not result in relevant diagnostic procedures.


Br. J. Cancer (1993), 67, 185–189 © Macmillan Press Ltd., 1993

### Hodgkin's disease diagnosed post mortem: A population based study

H. Hasle[1] & A. Mellemgaard[2]

[1]*Division of Haematology, Department of Oncology, Odense University Hospital, DK-5000 Odense C;* [2]*Danish Cancer Registry, Institute of Cancer Epidemiology, Box 839, DK-2100 Copenhagen, Denmark.*

**Summary** All cases of Hodgkin's disease (HD) notified to the Danish Cancer Registry from 1976 through 1987 in patients less than 70 years old were reviewed in order to identify patients in whom a correct diagnosis was established only post mortem. The case records of such patients were reviewed in a search for clinical features that could have ensured a correct pre mortem diagnosis.

HD was diagnosed after death in 31 patients in this unselected population based study and thus constituting only 2.4% of all patients less than 70 years with HD, but 14.1% of the group aged 65–69 years. Most patients were identified during the first part of the study period, which may reflect a decreasing autopsy rate. HD was considered to be a coincidental finding in four patients and the primary cause of death in 27 patients. Among the later 27 patients a number of unfavourable prognostic factors were a common finding: persistent unex-plained fever and weight loss, pencytopenia, hepatic involvement, bone marrow involvement, advanced stage disease, and lymphocytic depletion histology. However, most of the patients had no concurrent diseases and may have benefitted from a correct diagnosis and a potentially curative treatment.

The many uncommon features of HD together with the frequent findings of falsely negative chest X-ray, bone marrow examination, liver biopsy, and ultrasound contributed to the difficulty in diagnosis. In about 1/3 of the patients clinical findings suggestive of lymphoma did not result in relevant diagnostic procedures.

The evolution of management strategies for Hodgkin's disease (HD) during the past three decades has served as a model for the development of effective cancer treatment programs. With contemporary treatment 65 to 80% of all patients with HD will achieve a sustained complete remission (Nordentoft *et al.*, 1980; DeVita *et al.*, 1990).

The clinical stage and especially the tumour burden at presentation remain among the strongest prognostic factors (Hagemeister, 1988; Specht *et al.*, 1988). An early diagnosis is, therefore, essential. The most common presentation of HD is peripheral adenopathy (Ultmann *et al.*, 1966) but a diversity of other clinical features may be prominent which may result in a delayed diagnosis. In a study of 359 patients aged 40 to 79 years the time span between initial symptoms and diagnosis was about 20 weeks (Guinee *et al.*, 1991).

Cases have been reported of patients who died of HD but in whom the correct diagnosis was established only at autopsy (Korman *et al.*, 1979; Lefkowitch *et al.*, 1985; Sobrinho-Simoes *et al.*, 1983; Trewby *et al.*, 1979). Such patients are deprived of a potentially curative treatment.

We have reviewed a national based group of patients with HD to identify cases diagnosed post mortem. It was the purpose of the study to quantify this group of patients in a population based setting and to search for clinical features that could have resulted in an earlier diagnosis.

### Material and methods

The Danish Cancer Registry has since 1943 received notifications of malignant diseases from all clinical and pathological departments in the country. The notifications to the registry is supplemented by a scrutiny of all death certificates. The registry's coverage of cancer occurrence in Denmark is virtually complete (Storm, 1988).

This study was limited to persons less than 70 years old at the time of diagnosis because the prognosis of Hodgkin's disease in persons aged 70 and more is very poor (Nordentoft *et al.*, 1980), and the autopsy rate is significantly lower in older persons (Cocchi *et al.*, 1986; Kristensen & Bille, 1989).

Correspondence: H. Hasle, Department of Paediatrics, Odense University Hospital, DK-5000 Odense C, Denmark.
Received 20 December 1991; and in revised form 13 August 1992.

During the 12-year period from January 1, 1976 through December 31, 1987 a total of 1283 new cases of HD in patients resident in Denmark, and aged less than 70 years at the time of diagnosis were notified. The age distribution in 5-year age groups is shown in Figure 1.

For each case a date of diagnosis is registered in the Cancer Registry. This may be the accurate date of diagnosis or the date of the hospital admission that led to the diagnosis. In order to identify all patients in whom the diagnosis of HD was not established before death, we reviewed all cases in whom the period from the registered date of diagnosis to the date of death was less than 3 months.

The relevant case records from the clinical and pathological departments were reviewed. Characteristics concerning symptoms, clinical course, laboratory, and pathology findings were recorded. When serial measurements of laboratory data were performed the most abnormal value was recorded.

In 66 patients the period from the registered date of diagnosis to the date of death was less than 3 months. The case records of three patients could not be retrieved. A review of the available 63 case records revealed that in 32 patients the diagnosis of HD was known at the time of death. Accordingly, the study group consists of the remaining 31 patients in whom the diagnosis of HD was established after death.

**Figure1** Incidence of Hodgkin's disease in Denmark 1976–1987 from 0 to 70 years of age. Presented in 5-year age groups.

### Results

### Occurrence

The age distribution of the patients in whom the diagnosis of HD was established after death is shown in Figure 2. The youngest patient was 53 years old at the time of death. The number of unrecognised HD cases increased significantly with increasing age. The study group constituted 2.4% of all patients less than 70 years diagnosed during the same period, but 14.1% of the group aged 65–69 years. Twenty of the 31 patients were male. A similar male preponderance was found among the total group of HD patients. The majority of the cases (22 out of 31) occurred during the first half of the study period.

Four patients died of causes unrelated to HD. No HD related symptoms had been observed prior to death, and the autopsy disclosed only minor tumour burden corresponding to stage IA or IIIA. Theses cases are considered truly coincidential findings and will not be discussed in further details.

The rest of the paper deals with the 27 patients in whom symptomatic but unrecognised HD was the only or the major cause of death.

### Clinical data

Fifteen patients were seen in departments of internal medicine, four patients in departments of surgery, five patients in both types of departments, and four patients were also or exclusively seen in specialised departments of oncology or haematology.

Clinical data of the patients are summarised in Table I. Unexplained prolonged fever was experienced during a median time of 40 days (range 12–230) by 18 patients.

Peripheral lymphadenopathy was noted in nine patients, but resulted in biopsy in only two cases. In one patient an inguinal lymph node biopsy showed chronic lymphadenitis and in the other patient a supraclavicular lymph node biopsy was interpreted as metastatic anaplastic carcinoma.

Hepatomegaly was noted in 15 patients. Autopsy demonstrated hepatic involvement in ten of these cases. Liver biopsy was performed in five patients, who were all proven later to have hepatic involvement. In one patient the biopsy showed HD, but the patient died before the result of the microscopic study was available. None of the hepatic biopsies in the remaining four patients showed HD. However, granuloma formation was noted in three of the cases.

Splenomegaly was diagnosed in seven patients and autopsy demonstrated splenic involvement in all cases.

Laparotomy was performed in three patients. In one patient cholecystitis was suspected and freeze microscopy was suggestive of carcinoma; the patient died the day following surgery. In another patient a colon tumour was suspected and freeze microscopy was suggestive of lymphoma. However, the biopsy was later reinterpreted as carcinoma. The patient died before a new biopsy was obtained. In the last patient gastric cancer was suspected but no freeze microscopy was done. The patient died before the result of the final microscopic examination was available.

**Figure 2** Age distribution of the 31 patients in whom Hodgkin's disease was diagnosed post mortem.

**Table I** Symptoms and clinical findings in the 27 patients who died of unrecognised Hodgkin's disease

| | *Number* | *%* |
|---|---|---|
| Prolonged fever | 18 | 67 |
| Weight loss | 18 | 67 |
| Night sweats | 5 | 19 |
| Pruritus | 2 | 7 |
| Peripheral lymphadenopathy | 9 | 33 |
| Hepatomegaly | 15 | 56 |
| Splenomegaly | 7 | 26 |

### Laboratory data

Haemoglobin was below 10 g/100 ml in 15 (60% of evaluable patients). Auto-immune haemolytic anaemia was the presenting feature in three patients. The white cell count was below $4 \times 10^9 l^{-1}$ in ten (43% of evaluable patients). The platelet count was below $150 \times 10^9 l^{-1}$ in ten (59% of evaluable patients) and above $450 \times 10^9 l^{-1}$ in three patients. Pancytopenia was noted in eight patients.

Bone marrow examination was performed in 12 patients at a median of 20 days (range 6–118) before the time of death. In five patients both aspirate and trephine biopsy was done. The bone marrow study was suggestive of HD in three patients, but no definite diagnosis was made. Myelofibrosis was demonstrated in two patients. No signs of malignancy were noted in the remaining bone marrow examinations. Autopsy demonstrated bone marrow involvement in ten of the 12 patients with a prior bone marrow examination.

Marked elevation of the erythrocyte sedimentation rate ($>50$ mm $h^{-1}$) was found in 14 of 22 patients.

Lactate dehydrogenase (LDH) was elevated in ten patients. Alanine or aspartate aminotransferase were elevated in 11 patients; eight of these later showed hepatic involvement. Bilirubin was elevated in six patients; five of these later showed hepatic involvement. Serum copper was measured only in one patient and found to be normal.

### Radiology data

Chest X-ray was done in 26 patients, at a median of 10 days (range 1–132) from last X-ray to death. Enlarged lymph nodes in the mediastinum or pulmonary hilum were noted in only two patients. However, involvement in the hilum or mediastinum was found at autopsy in 15 of the 26 patients. Pulmonary abnormalities suggestive of primary pulmonary cancer or metastases were seen in three patients. CT-scan of the thorax was performed in one patient and considered to be without abnormalities. At autopsy in the same patient one month later enlarged lymph nodes in the mediastinum were disclosed.

Ultrasound of the abdomen was performed in five patients at a median of 30 days (range 1–70) before death. Hepatomegaly was noted in two and splenomegaly in one patient. Enlarged lymph nodes were not seen on ultrasound in any of the five patients, but all five patients had involvement of the retroperitoneum at autopsy. Two of the five patients were also examined with CT-scan of the abdomen 20 and 42 days before death respectively. In both patients enlarged lymph nodes in the retroperitoneum were noted, but biopsy was not performed.

### Pathology data

Autopsy was performed in all patients but one. The diagnosis in the later patient was based on the findings from a liver biopsy obtained one day before death.

Histopathologic classification showed lymphocytic predominance in four patients, nodular sclerosis in one patient, mixed cellularity in four patients, and lymphocytic depletion in 12 patients. Six cases were unclassified. Review of the histologic diagnoses was not achieved.

According to the Ann Arbor classification one patient had stage I (the patient was admitted because of dyspnea, but was dead on arrival at the hospital. Autopsy demonstrated cervical HD compressing the larynx). The stages IIB, IIIB, and IVA were found in one patient each. The remaining 23 patients were all classified as stage IVB.

Disease extent based on autopsy findings is shown in Table II. The numbers must be considered as minimal figures since in a considerable number of cases information from the autopsy report was incomplete. This was especially true for peripheral lymph nodes, bone marrow, and spleen for which information was lacking in 12, seven and four cases respectively. Involvement of extralymphatic sites outside bone marrow and liver occurred in 12 patients (44%).

*Reasons for failure of diagnosis*

After a thorough review of all case records, we have classified the patients into five groups according to what we considered to be the main reason why the correct diagnosis was not established before death. The result is shown in Table III.

The median time span from first symptom to first consultation at a general practitioner could be evaluated in seven patients and was 30 days (range 14–135). The median time for the same patients from first consultation to admission in hospital was 10 days (range 0–60). In 17 patients the median time from first symptom to admission could be estimated and was 55 days (range 2–150). The median time from admission to death in the 27 patients was 31 days (range 0–450). Three patients were admitted too terminally ill for diagnostic procedures. About 1/3 of the patients showed symptoms which were definitely suggestive of lymphoma, but the relevant diagnostic procedures were not undertaken. It is especially noteworthy that lymphadenopathy (peripheral or in the retroperitoneum) was observed in nine patients without attempts of biopsy. Patients without peripheral lymphadenopathy or visible abnormalities on chest X-ray, often combined with falsely negative liver biopsy or bone marrow examination, remained an enigma to the clinicians.

Most of the patients had no concurrent diseases. Two patients were mentally handicapped, which in one case was the reason for diagnostic passivity. Three patients were alcoholics but active diagnostic procedures were performed in all of them. Five patients had other severe somatic diseases; apoplexia, diabetes mellitus with complications, adipositas, chronic bronchitis, and arteriosclerosis. None of the patients suffered from psychiatric disease. None of the patients were reluctant to the proposed investigations.

**Table II** Localisation of HD as demonstrated at autopsy in 26 of the 27 patients who died of unrecognised Hodgkin's disease

| | *Number* | *%* |
|---|---|---|
| Peripheral lymph nodes | 8 | 30 |
| Pulmonary hilum/mediastinum | 15 | 56 |
| Retroperitoneum | 23 | 85 |
| Spleen | 17 | 63 |
| Liver | 16 | 59 |
| Bone marrow | 13 | 48 |
| Lung | 4 | 15 |
| Stomach | 3 | 11 |
| Kidney | 3 | 11 |
| Soft tissue | 2 | 7 |
| Other sites (trachea, heart, colon, pancreas, peritoneum, bone) | 6 | 22 |

### Discussion

In this unselected population based study we found that unrecognised HD constituted 2.4% in patients less than 70 years of age, but the frequency increased significantly with increasing age. The study by Nordentoft *et al.* comprising all age groups in the same geographic area from 1971 to 1979 identified 16 patients (2.1%) at autopsy only (Nordentoft *et al.*, 1980). A remarkably high frequency was found in a study from Stockholm in which unrecognised HD was found at autopsy in 31 elderly patients who represented 12% of all patients aged 15 years and more (Wedelin *et al.*, 1984).

Most of the cases in our study occurred during the first half of the study period. A possible explanation could be the better access to CT-scan and ultrasound during recent years which may facilitate a prompt diagnosis. However, the four cases considered as coincidental findings all occurred in 1977 and 1978, and the frequency of HD found at autopsy during an earlier period in the same area was lower (Nordentoft *et al.*, 1980). Therefore, another possible explanation of the observed trend of a decreasing frequency of unrecognised HD could be a falling autopsy rate (Cocchi *et al.*, 1986; Kristensen & Bille, 1989). It has previously been shown that physicians are often unable to identify from the clinical data autopsies likely to have a high diagnostic yield (Bekker & Jensen, 1986; Landefeld *et al.*, 1988), and the agreement between the clinical and pathological diagnosis is lowest among the oldest patients (Cocchi *et al.*, 1986).

Pre-mortem biopsy was in three cases interpreted as carcinoma and in one case as chronic lymphadenitis. These mistakes are presumably reduced with modern immunohistologic methods.

Lymphocytic depletion (LD) is seen in 5% of unselected

**Table III** Reasons why the diagnosis of HD was not established before death, with number of days from first admission to death

| | | *Patients* | *Days* | |
|---|---|---|---|---|
| | | *(n = 27)* | *Median* | *Range* |
| I | Patient admitted terminally ill | 3 | 5 | 0–12 |
| II | Lack of essential investigations or unacceptable long time spent waiting for examinations to be performed, or for results of performed examination | 10 | 33 | 4–450 |
| III | Diagnostic passivity because of the somatic or mental condition of the patient | 3 | 13 | 11–132 |
| IV | Diagnostic passivity because of wrongly suspected somatic illness[a] | 6 | 56 | 9–120 |
| V | Diagnosis not obtained in spite of relevant active approached | 5 | 38 | 14–78 |

Note:
[a]Two suspected of pulmonary cancer, one considered as progression of known CLL, one with a lymph node biopsy suggestive of adenocarcinoma, and one considered to be primary myelofibrosis. One case was considered as cholangitis.

Danish patients (Nordentoft *et al.*, 1980) but in the present series LD represented 57% of all cases with a definite histologic classification. LD is associated with higher age, advanced stage, poor prognosis, and frequently occurs subdiaphragmatic without peripheral lymphadenopathy (Neiman *et al.*, 1973). The clinical features of LD frequently lead to considerable difficulty in diagnosis (Neiman *et al.*, 1973). The histopathologic diagnosis of HD is recognised to be a difficult area in pathology, especially regarding the distinction of the LD subtype from non Hodgkin's lymphoma (NHL). Reviews of cases of LD frequently result in reclassification to other types of HD or to NHL (Kant *et al.*, 1986). It is possible that some of the cases in the present study represent NHL, extending the problem to unrecognised lymphomas.

Fever was a prominent feature in the majority of the patients. The definition of fever of unknown origin (FUO) with a temperature above 38.3°C during a three week period without any definite diagnosis after one week in hospital was fulfilled by 14 patients (52%). In larger series of FUO a neoplastic cause, most often lymphoma, was identified in about 30% of the cases (Howard *et al.*, 1977; Larson *et al.*, 1982). A neoplastic cause is most likely among patients above 40 years of age (Deal, 1971).

Subdiaphragmatic disease occurred in all but four of the patients in the present series and is often associated with older age, advanced stage, and LD (Krikorian *et al.*, 1986; Villamor *et al.*, 1991). Such patients frequently present with systemic symptoms, e.g. FUO, without symptoms of an abdominal mass (Krikorian *et al.*, 1986). In such cases abdominal ultrasound or CT-scan should be performed to search for retroperitoneal masses. However, as shown in the present study the retroperitoneum may be difficult to visualise with ultrasound.

Hepatic involvement was a frequent finding in this series. Liver involvement, often with a presumptive diagnosis of primary liver disease, has also been prominent in other cases of HD diagnosed post mortem (Lefkowitch *et al.*, 1985, Sobrinho-Simoes *et al.*, 1983; Trewby *et al.*, 1979). Hepatic involvement is seen in only 6% of unselected patients with HD (Nordentoft *et al.*, 1980) but is frequently reported in association with LD (Neiman *et al.*, 1973). Liver biopsy was not conclusive in four out of five cases in which it was performed. However, granuloma formation was observed in three patients which may be suggestive of HD (Brincker, 1986).

In the present series bone marrow involvement was demonstrated in 48% in contrast with 5 to 15% in other series (Jacquillat *et al.*, 1981; Myers *et al.*, 1974; O'Carroll *et al.*, 1976). Bone marrow involvement is often present in cases of LD (Neiman *et al.*, 1973). Of the patients with bone marrow involvement 77% had a bone marrow examination performed without establishment of the correct diagnosis. The bone marrow biopsy from two patients demonstrated myelofibrosis, a recognised presenting feature of HD (Meadows *et al.*, 1989). In some cases only aspirates were performed which are usually insufficient for lymphoma diagnosis (Ferrant *et al.*, 1975; Neiman *et al.*, 1973).

We found a remarkably high frequency (44%) of involvement of extralymphatic sites outside the bone marrow and liver. This is unusual even in cases of LD with widespread disease (Neiman *et al.*, 1973). This uncommon feature of HD may have contributed to the difficulty in diagnosis.

The 5 million inhabitants of Denmark are all covered by the social security system and have free access to a general practitioner and hospital treatment. Accordingly a delayed diagnosis should not occur because of a lack of medical services. However, three patients were admitted in hospital too ill of their HD for diagnostic procedures. None of them had sought medical advice before the day of admission.

In most patients the time span from first symptom or admission to death was long enough to allow a number of investigations to be performed. The frequent findings of falsely negative chest X-ray, bone marrow examination, liver biopsy, and ultrasound were misleading in many cases. In about 1/3 of the cases clinical findings did not lead to relevant diagnostic procedures (e.g. the lack of biopsy of unexplained peripheral lymphadenopathy, search for lymphoma in cases of FUO).

Overall unrecognised HD represented only a minority of all HD cases, but a significant number of older patients. Most of the patients had a number of unfavourable prognostic factors, but since most of them did not have any concurrent diseases they may have benifitted from a correct premortem diagnosis and a potentially curative treatment.

### References

BEKKER, C. & JENSEN, N.K. (1986). Unexpected findings at autopsy. (Original in Danish, Engl abstr). *Ugeskr. Laeger*, **148**, 3199–3202.

BRINCKER, H. (1986). Sarcoid reactions in malignant tumours. *Cancer Treat. Rev.*, **13**, 147–156.

COCCHI, A., VECCHIO, F.M., PAHOR, M., ANTICO, L., FRANCESCHINI, G., FARINA, G. & CARBONIN, P.U. (1986). Autopsy rate in younger and older hospitalized patients. *Eur. J. Epidemiol.*. **2**, 151–157.

DEAL, W.B. (1971). Fever of unknown origin. Analysis of 34 patients. *Postgrad. Med.*, **50**, 182–188.

DEVITA, V.T., HUBBARD, S.M. & LONGO, D.L. (1990). Treatment of Hodgkin's disease. *J. Natl Cancer Inst. Monogr.*, **10**, 19–28.

FERRANT, A., RODHAIN, J., MICHAUX, J.L., PIRET, L., MALDAGUE, B. & SOKAL, G. (1975). Detection of skeletal involvement in Hodgkin's disease: A comparison of radiography, bone scanning, and bone marrow biopsy in 38 patients. *Cancer*, **35**, 1346–1353.

GUINEE, V.F., GIACCO, G.G., DURAND, M., VAN DEN BLINK, J.W., GUSTAVSSON, A., MCVIE, J.G., ZEWUSTER, R., DISCHE, S., FAHEY, T. & LANE, W. (1991). The prognosis of Hodgkin's disease in older adults. *J. Clin. Oncol.*, **9**, 947–953.

HAGEMEISTER, F.B. (1988). Prognostic factors in decision-making in the clinical management of Hodgkin's disease. *Hematol. Oncol.*, **6**, 257–269.

HOWARD, P., HAHN, H.H., PALMER, R.L. & HARDIN, W.J. (1977). Fever of unknown origin: a prospective study of 100 patients. *Tex. Med.*, **73**, 56–59.

JACQUILLAT, C., AUCLERC, G., AUCLERC, M.F., ANDRIEU, J.M., WEIL, M. & BERNARD, J. (1981). Hodgkin's disease: Characteristics and prognosis of forms with initial bone marrow involvement. *Nouv. Presse Med.*, **10**, 95–100.

KANT, J.A., HUBBARD, S.M., LONGO, D.L., SIMON, R.M. DEVITA, V.T. & JAFFE, E.S. (1986). The pathologic and clinical heterogeneity of lymphocyte-depleted Hodgkin's disease. *J. Clin. Oncol.*, **4**, 284–294.

KORMAN, L.Y., SMITH, J.R., LANDAW, S.A. & DAVEY, F.R. (1979). Hodgkin's disease: Intramedullary phagocytosis with pancytopenia. *Ann. Intern. Med.*, **91**, 60–61.

KRIKORIAN, J.G., PORTLOCK, C.S. & MAUCH, P.M. (1986). Hodgkin's disease presenting below the diaphragm: A review. *J. Clin. Oncol.*, **4**, 1551–1562.

KRISTENSEN, F.B., & BILLE, H. (1989). Decreasing rate of autopsies in Denmark. (Original in Danish). *Ugeskr, Laeger*, **151**, 2899–2900.

LANDEFELD, C.S., CHREN, M.M., MYERS, A., GELLER, R., ROBBINS, S. & GOLDMAN, L. (1988). Diagnostic yield of the autopsy in a university hospital and a community hospital. *N. Engl. J. Med.*, **318**, 1249–1254.

LARSON, E.B., FEATHERSTONE, H.J. & PETERSDORF, R.G. (1982). Fever of undetermined origin: Diagnosis and follow-up of 105 cases, 1970–1980. *Medicine*, **61**, 269–292.

LEFKOWITCH, J.H., FALKOW, S. & WHITLOCK, R.T. (1985). Hepatic Hodgkin's disease simulating cholestatic hepatitis with liver failure. *Arch. Pathol. Lab, Med.*, **109**, 424–426.

MEADOWS, L.M., ROSSE, W.R., MOORE, J.O., CRAWFORD, J., LASZLO, J. & KAUFMAN, R.E. (1989). Hodgkin's disease presenting as myelofibrosis. *Cancer*, **64**, 1720–1726.

MYERS, C.E., CHABNER, B.A., DEVITA, V.T. & GRALNICK, H.R. (1974). Bone marrow involvement in Hodgkin's disease: Pathology and response to MOOP chemotherapy. *Blood*, **44**, 197–204.

NEIMAN, R.S., ROSEN, P.J. & LUKES, R.J. (1973). Lymphocyte-depletion Hodgkin's disease. *N. Engl. J. Med.*, **288**, 751–755.

NORDENTOFT, A.M., PEDERSEN-BJERGAARD, J., BRINCKER, H., ANDERSEN, E., PEDERSEN, M., NIELSEN, J.B., JENSEN, K.B., NISSEN, N.I., JENSEN, T.S., VIDEBÆK, A., JENSEN, M.K. & WALBOM-JØRGENSEN, S. (1980). Hodgkin's disease in Denmark. A national clinical study by the Danish Hodgkin study group, LYGRA. *Scand. J. Haematol.*, **24**, 321–334

O'CARROLL, D.I., MCKENNA, R.W. & BRUNNING, R.D. (1976). Bone marrow manifestations of Hodgkin's disease. *Cancer*, **38**, 1717–1728.

SOBRINHO-SIMOES, M., PAIVA, M.E., GONCALVES, V., SALDANHA, C., SALEIRO, J.V. & SERRAO, D. (1983). Hodgkin's disease with predominant infradiaphragmatic involvement and massive invasion of the bone marrow. A necropsic study of nine cases. *Cancer*, **52**, 1927–1932.

SPECHT, L., NORDENTOFT, A.M., COLD, S., CLAUSEN, N.T. & NISSEN, N.I. (1988). Tumor burden as the most important prognostic factor in early stage Hodgkin's disease. *Cancer*, **61**, 1719–1727.

STORM, H.H. (1988). Completeness of cancer registration in Denmark 1943–1966 and efficacy of record linkage procedures. *Int. J. Epidemiol.*, **17**, 44–49.

TREWBY, P.N., PORTMANN, B., BRINKLEY, D.M. & WILLIAMS, R. (1979). Liver disease as presenting manifestation of Hodgkin's disease. *Q.J. Med.*, **48**, 137–150.

ULTMANN, J.E., CUNNINGHAM, J.K. & GELLHORN, A. (1966). The clinical picture of Hodgkin's disease. *Cancer Res.*, **26**, 1047–1060.

VILLAMOR, N., REVERTER, J.C., MARTI, J.M., MONTSERRAT, E. & ROZMAN, C. (1991). Clinical features and response to treatment of infradiaphragmatic Hodgkin's disease. *Eur. J. Haematol.*, **46**, 38–41.

WEDELIN, C., BJÖRKHOLM, M., BIBERFELD, P., HOLM, G., JOHANSSON, B. & MELLSTEDT, H. (1984). Prognostic factors in Hodgkin's disease with special reference to age. *Cancer*, **53**, 1202–1208.